# Acquired platelet disorders

**DOI:** 10.3389/fmed.2025.1638994

**Published:** 2025-11-11

**Authors:** Rahaf Mahmoud Altahan

**Affiliations:** 1 Hematology Unit, Pathology and Clinical Laboratory Medicine Administration, King Fahad Medical City, Riyadh Second Health Cluster, Riyadh, Saudi Arabia; 2 College of Medicine, Alfaisal University, Riyadh, Saudi Arabia

**Keywords:** acquired platelet defects, acquired platelet disorders, immune thrombocytopenia, acquired coagulation disorders, approach to acquired bleeding disorders, therapy of acquired bleeding disorders

## Abstract

Platelets are essential to primary hemostasis, and defects in their number or function can lead to clinically significant bleeding or thrombosis. Acquired platelet disorders are far more common than the inherited forms and arise in a wide range of settings, including drug exposure, autoimmune, systemic, and critical illnesses. This review examines current understanding of the mechanisms driving acquired platelet dysfunction and outlines the diagnostic and therapeutic approaches that are shaping contemporary standards of care. Drug-related and immune-mediated platelet defects remain the most recognized causes in clinical practice. Nevertheless, increasing evidence points to inflammation, particularly the profound dysregulation seen in sepsis, as a major contributor to abnormal platelet behavior. Although sepsis-associated platelet defects are frequent in practice and correlate with organ injury and adverse outcomes, they are rarely acknowledged in traditional frameworks of “acquired platelet disorders,” which tend to focus on classic hematologic and pharmacologic causes. This review summarizes current evidence on acquired platelet disorders and emphasizes the clinical and pathophysiologic relevance of sepsis-induced platelet dysfunction.

## Introduction

Platelets play a crucial role in primary hemostasis. Platelet defects can be congenital or acquired, with the latter being far more common. These disorders are broadly categorized into quantitative abnormalities, defined as a platelet count below the lower limit of the normal range (<150 × 10^9^/L). Thrombocytopenia is further classified by severity: mild (100–149 × 10^9^/L); moderate (50–99 × 10^9^/L); and severe (<50 × 10^9^/L), and qualitative, where the platelet function is defective. In many settings, these two conditions can coexist: i.e., a drop in platelet count accompanied by abnormal platelet function. Platelet defects usually result in mucocutaneous bleeding (e.g., petechiae, purpura, epistaxis, gingival bleeding, and menorrhagia). In this article, the pathophysiology of the most encountered conditions in clinical practice that lead to acquired platelet dysfunction will be discussed. A special focus will be given to the role of inflammation in platelet dysfunction, more accurately, platelet activation, as this is a rapidly evolving field that remains under-recognized in conventional classifications of acquired platelet disorders.

## Categories of acquired platelet disorders

Acquired platelet disorders are far more common than congenital platelet defects. Causes include systemic diseases, medications, antibodies, and other factors that can negatively impact platelets. This detrimental effect is typically associated with the underlying cause, and in most cases, removing the causative agent (i.e., discontinuing the causative drug or treating the underlying disease) leads to platelet recovery. However, in patients with chronic diseases, platelet defects are more challenging and their defects are commonly confounded by disturbances in other related systems (e.g., defects in the coagulation and fibrinolysis systems).

## Drug-induced

Drugs are among the most common causes of acquired platelet dysfunction in clinical practice. Nearly any drug has the potential to induce platelet dysfunction, either quantitative (thrombocytopenia) or qualitative (functional impairment), through diverse pathological mechanisms. Both forms of dysfunction are clinically significant due to their impact on hemostasis. Drug-induced platelet dysfunction can be broadly categorized into two groups: (1) medications that intentionally target platelet function, such as antiplatelet agents, and (2) drugs prescribed for unrelated indications that exert off-target effects on platelets.

• Antiplatelet drugs: Antiplatelet medications are taken with the intent to suppress platelet function, as a therapeutic modality for patients with cardiovascular diseases, myeloproliferative disorders, etc., and are in fact among the most commonly prescribed drugs (1).

o Aspirin (Acetylsalicylic acid) is among the most commonly used antiplatelet therapies. Initially introduced in the late 1890s, it was first used as an anti-inflammatory drug, and nearly 70 years later, its role in platelet inhibition was recognized (2). Aspirin primarily exerts its action by irreversibly inhibiting cyclooxygenase-1 (COX-1), an enzyme essential for converting arachidonic acid to thromboxane A2 (TXA_2_), a potent promoter of platelet aggregation. By inhibiting COX-1, the formation of TXA_2_ is reduced, thereby impairing the amplification of platelet aggregation (3). Of note, aspirin also irreversibly inhibits COX-2, another isoform of the COX enzyme that is involved in the production of prostacyclin (PGI_2_), an inhibitor of platelet aggregation. However, COX-2 inhibition is less pronounced and does not significantly contribute to the antiplatelet effect of the drug (4, 5). Due to the irreversible action of aspirin on platelets, its effect lasts for the lifespan of platelets *in vivo*, which is approximately 7–10 days. However, when accounting for the production of new platelets (around 10^11^ per day), its effect persists for up to 5 days (4, 6).
αIIbβ3 antagonists abciximab, tirofiban, and eptifibatide are the most potent antiplatelet therapy that inhibit integrin αIIbβ3, significantly impairing platelet aggregation induced by all platelet activators. They are administered intravenously. While the functional inhibition of eptifibatide and tirofiban lasts for about 4–8 h due to the reversible binding to the αIIbβ3 integrin, abciximab’s inhibitory effect can last up to 48 h due to the irreversible integrin binding (3). In addition to the functional platelet inhibition, thrombocytopenia is a rare but serious complication of these drugs that can develop either acutely or delayed, occurring within 24 h or up to 14 days of drug administration, respectively. Platelet nadir ranges from mild to profound, with the profound being rare and occurring in less than 1% of patients with the first administration. However, the risk increases with drug re-administration. This complication is immune-mediated, as it develops due to antibodies that recognize the integrin in the presence of these drugs. Studies showed that binding of eptifibatide and tirofiban causes a significant conformational change and potentially creates a neoepitope. Additionally, the pathological antibodies recognize the neoepitope only in the presence of the drug (7). Zalunfiban, a novel, second generation, short-acting, αIIbβ3 antagonist administered subcutaneously, is being explored as a prehospital treatment for ST-elevation myocardial infarction patients intended for primary percutaneous coronary intervention, due to its potent platelet inhibition and rapid onset of action, making it an ideal candidate for early intervention (7). Although the dependence of antibody binding on the conformational change is still not confirmed, future studies on zalunfiban are expected to give some evidence, as this drug does not cause receptor conformation change, and could, in theory, have a lower incidence of thrombocytopenia compared to the other drugs.
Other antiplatelet medications, including P2Y₁₂ receptor inhibitors, are summarized in Table 1. While these agents offer substantial clinical benefits, particularly in the prevention of thrombotic complications, they are associated with a significantly increased risk of bleeding. Gastrointestinal bleeding remains the most frequent site of major hemorrhage. This risk is further heightened in the presence of compounding factors, such as concomitant dual antiplatelet therapy (e.g., post-stent placement), advanced age, underlying bleeding disorders, or concurrent use of anticoagulants (8).

• Drugs that inadvertently impair platelet function: An extended list of drugs that are taken for a variety of indications can cause a variable degree of platelet dysfunction, and their bleeding manifestations can vary from minor bruising and mucocutaneous bleeding to life-threatening hemorrhages, especially in patients with underlying hemostatic defects (9). The most classic example is non-steroidal anti-inflammatory drugs (NSAIDs) other than aspirin. These drugs, like ibuprofen, naproxen, and indomethacin, reversibly inhibit COX-1 and accordingly cause a short-term platelet defect lasting around 24 h (10). A list of drugs that inadvertently inhibit platelets is summarized in Table 1.

**Table 1:** List of antiplatelet medications with their mechanism of platelet inhibition and approved use.

Drug class	Antiplatelet or inadvertent PLT inhibition	Mechanism of platelet inhibition	Examples	Approved use
COX-1 Inhibitors NSAIDs	Aspirin: antiplatelet Other NSAIDs: inadvertent PLT Inh.	COX-1 inhibition leads to a reduction in TXA_2_ formation, impairing platelet aggregation	Irreversible: aspirin Reversible: Ibuprofen, Naproxen, and Indomethacin	(Mild to moderate) Pain relief Inflammatory conditions, and fever reduction Also, for aspirin (only): secondary prevention of cardiovascular/cerebrovascular event, post-PCI, ACS
P2Y_12_ inhibitors	Antiplatelet	P2Y_12_ receptor inhibition blocks ADP-induced platelet aggregation	Irreversible, prodrug (Thienopyridines): Clopidogrel and Prasugrel Reversible, active: Ticagrelor	ACS, post-PCI, secondary prevention of ischemic events
αIIbβ3 antagonists	Antiplatelet	Inhibition of integrin αIIbβ3 significantly impair platelet aggregation	Abciximab, tirofiban, and eptifibatide	ACS, percutaneous cardiac interventions
Tricyclic antidepressants	Inadvertent PLT Inh.	Evidence suggests interference with serotonin uptake	Amitriptyline, nortriptyline	Depression, anxiety, and certain types of pain
SSRIs (Antidepressants)	Inadvertent PLT Inh.	Inhibit serotonin and norepinephrine reuptake; reduce platelet serotonin content needed for aggregation	Fluoxetine, Sertraline	Depression and anxiety disorders
Dipyridamole	Antiplatelet	Inhibits PDE and adenosine uptake (increasing extracellular adenosine) PDE inhibition prevents the degradation of cAMP, while adenosine raises its intracellular levels by activating platelet A_2A_ adenosine receptors Leading to cAMP elevation and inhibition of platelet aggregation (133)	Aggrenox long-acting dipyridamole/aspirin combination	Secondary stroke prevention
Cilostazol	Antiplatelet		–	Intermittent claudication associated with PAD
Dextran (plasma volume expander)	Inadvertent PLT Inh.	Direct interference with platelet aggregation due to dextran’s effect on blood viscosity; rheological effects and interference with platelet-fibrin interaction	–	Expanding plasma volume, in cases of shock or blood loss
Beta-blockers	Inadvertent PLT Inh.	Inhibition of adrenergic stimulation by blocking β-adrenergic receptors. Interfere with platelet membrane functions (e.g., receptor expression and signaling) due to their local anesthetic or membrane-stabilizing properties Reduced TXA_2_ synthesis and aggregation^‡^	Metoprolol, atenolol, propranolol	Hypertension, heart failure, and arrhythmias
Calcium channel blockers	Inadvertent PLT Inh.	Inhibition of calcium influx into platelets affecting mainly serotonin-induced PLT aggregation (134)*	Amlodipine, verapamil	hypertension, angina, and arrhythmias
Beta lactam antibiotics	Inadvertent platelet Inh.	TP: (immune-mediated): drug-dependent antibodies bind to PLT glycoproteins, leading to destruction in the RES-mainly the spleen PLT function defects: inhibition of aggregation, granule secretion and interactions with VWF	Penicillin, cephalosporins	Bacterial infections
Nitrates (vasodilators)	Inadvertent platelet Inh.	Increase in cGMP levels by increasing NO (134)*	Nitroglycerin, isosorbide dinitrate	Heart failure management
Proteasome inhibitors	Inadvertent platelet Inh.	Impaired platelet activation and reduced aggregation Inhibition of NF-κB signaling; downregulation of activation markers (e.g., P-selectin, αIIbβ3); altered megakaryopoiesis (135)	Bortezomib, carfilzomib	MM, MCL
Valproic acid	Inadvertent platelet Inh.	TP in up to 54% of patients (136), inhibition of the COX pathway, leading to decreased synthesis of THXA_2_, a potent promoter of platelet aggregation	–	Epilepsy, bipolar disorders, migraine prophylaxis

ACS, acute coronary syndrome; ADP, adenosine diphosphate; cAMP, cyclic AMP; COX-1, cyclooxygenase-1; GPCR, G protein-coupled receptor; Inh, inhibition; MCL, mantle cell lymphoma; MM, multiple myeloma; NSAIDs, non-steroidal anti-inflammatory drugs; PAD, peripheral artery disease; PCI: percutaneous coronary intervention; PDE3, phosphodiesterase type 3; PLT, platelets; SSRIs, selective serotonin reuptake inhibitors; RES, reticuloendothelial system; TP, thrombocytopenia; TXA_2_, thromboxane A2; VWF, von Willebrand factor.

^*^Uncertain clinical impact.

^‡^Preclinical research.

## Herbals, supplements and alcohol-induced

• Herbal medicines are commonly used, but their use is often overlooked in routine care, with the World Health Organization estimating that 80% of the global population uses such preparations (11). This practice is widespread, particularly in regions like the Middle East (12). It is important to note that most of the current evidence regarding the effects of herbal medications on platelet aggregation is preclinical, with some noted inconsistencies in the studies. This variability can be attributed to several factors, including inconsistent laboratory findings, potential biases in some clinical trials, and differences in study populations (e.g., patients versus healthy individuals). Given these uncertainties, the clinical benefit of these herbal therapies, particularly in the context of antithrombotic effects, remains questionable. As such, until more robust, well-controlled studies are conducted to clarify their efficacy and safety, these herbal treatments should not be recommended for patients with an increased risk of bleeding or those concurrently using anticoagulants. The potential risks, including an elevated risk of bleeding, currently outweigh any unproven benefit (3, 13). *Ginkgo biloba* extract (GBE) has been extensively studied for its potential benefits in cognitive function, revascularization, and anxiety. Studies showed a rapid platelet inhibition effect, mainly by inhibiting arachidonic acid-induced aggregation; this inhibition shows a synergistic effect when combined with aspirin (12). A summary of some of the herbs used, along with their antiplatelet effects, is presented in Table 2.
Alcohol consumption is linked to both platelet inhibition and activation. The unified theory behind this is that in alcohol consumption, platelets are partially activated with subsequent partial platelet degranulation, leading to platelet exhaustion and dysfunction. Starting with platelet inhibition, aggregation to several agonists is impaired in the presence of ethanol. This was confirmed in several studies, with the earliest conducted by Haut and Cowan (14). Additionally, the mobilization of arachidonic acid from phospholipid membranes is impaired, resulting in reduced thromboxane A2 (TXA_2_) generation (15). Furthermore, ethanol inhibits the expression of αIIbβ3, reducing fibrinogen binding and subsequent aggregation. These effects usually occur within 10 min of ingestion and are dose-dependent, with higher concentrations of ethanol exerting more pronounced inhibitory effects (16). On the other hand, platelet activation occurs mainly due to a direct effect of ethanol, which transiently lowers intraplatelet cyclic adenosine monophosphate (cAMP), an inhibitory second messenger (see the section “Liver-associated platelet dysfunction” for details). This reduction removes cyclic nucleotide restraint on platelet signaling, facilitating downstream activation. This is evident by increased expression of the surface P-selectin, formation of platelet–neutrophil complexes that promote neutrophil extracellular traps and amplify inflammation (17, 18), and increased platelet aggregation with thrombin, when blood samples are taken beyond 20 min of alcohol ingestion (19).
  The shifts in platelet states from reduced to increased platelet aggregation after alcohol ingestion suggest a transition from a hypocoagulable to a hypercoagulable state (19) and might partly explain the sudden death associated with withdrawal or binge drinking and the predisposition of individuals to different bleeding patterns, like upper GI bleeding and hemorrhagic stroke (20).
  Overall, these findings indicate that alcohol has complex, dose- and time-dependent effects on platelet function. Its impact can be inhibitory or activating, depending on the pathway involved and the timing of ingestion. For instance, inhibition may predominate shortly after intake, whereas activation may emerge later (20). Ethanol has an additional significant impact on coagulation factors and the fibrinolysis system, further exacerbating platelet dysfunction in these patients, which extends beyond the scope of this review (21).
  Besides the platelet activity dysfunction, thrombocytopenia (TP) can also be seen, especially with heavy alcohol consumption. Its severity correlates with the extent of alcohol ingestion “intoxication” and, in general, is a quickly transient condition, with rebound thrombocytosis during 1–3 weeks of cessation, followed by subsequent normalization (22). The pathophysiology of TP is mainly due to myelosuppression, direct platelet toxicity in the bloodstream, and possibly increased platelet apoptosis (23). Reduced thrombopoietin production by hepatocytes also contributes to TP; this may result from liver injury or nutritional deficiencies (24). It is suggested that genetic alterations affect the impact of alcohol-induced TP. This was demonstrated by Yokoyama et al., who showed that variations in Aldehyde Dehydrogenases ALDH2 and 1B genotypes markedly affect both the degree of TP and the trajectory of platelet recovery in individuals with alcohol dependence (25). Finally, the mean platelet volume (MPV) is commonly elevated in alcohol consumption. The proportion of reticulated “young” platelets is relatively increased in individuals with alcohol consumption, leading to an increase in the MPV, a commonly observed finding in this condition. This is likely a compensation mechanism for the peripheral destruction resulting from direct damage (22).

**Table 2:** List of herbs that impair platelet function with their mechanism of platelet inhibition.

Herbs/supplements	Mechanism of PLT impairment
Garlic	AGE inhibit PLT aggregation by increasing cAMP and cGMP and preventing fibrinogen binding to the αIIbβ3 receptor (137)
Ginger (*Zingiber officinale*)	Variable effect: from no significant effect on PLT aggregation (by ADP and epi) (138) to reduced PLT aggregation with epinephrine only (139) to reduced PLT aggregation and inhibition of TXB_2_ production* (140)
Green tea^¶^	Reduced platelet aggregation (13, 141)
*Cinnamomum cassia*	Reduced platelet aggregation (142)^‡^
Dong Quai (Angelica sinensis)	Contains coumarin derivatives that reduce rate of platelet aggregation, thrombin-induced platelet activation, and TXB_2_* production (143)
Feverfew (*Tanacetum parthenium*)	Reduced platelet aggregation and serotonin release (13)
Turmeric (*Curcuma longa*)	Reduced GPVI-mediated platelet activation and dense granule secretion (144)^‡^
Asian Ginseng (*Panax ginseng*)	Reduced platelet aggregation, reduced granule secretion, calcium ion mobilization, and activation of integrin αIIbβ3, increase cAMP (96, 145)^‡^
American Ginseng (*Panax quinquefolius*)	Synergistic effect in platelet inhibition when combined with DAPT (146)^‡^
Willow (*Salix alba*)	Reduced AA- induced platelet aggregation (147)
Black Tree Fungus (*Auricularia polytricha*)	Reduced ADP-induced platelet aggregation (148)^‡^
Cannabis	Two active metabolites with opposing effects: THC and CBD THC can activate platelets by increasing the expression of glycoprotein αIIbβ3 and P-selectin (149) CBD reduce platelet aggregation (150)

AA, arachidonic acid; AGE, Aged garlic extract; CBD, cannabidiol; COX, Cyclooxygenase; DAPT, dual antiplatelet therapy; PLT, platelets; THC, tetrahydrocannabinol; TXA_2_, thromboxane A2; TXB_2_, thromboxane B_2_.

^*^Often used as a biomarker for assessing TXA_2_ formation, as its presence in the blood reflects TXA_2_ production.

^‡^Preclinical research.

^¶^Matcha, a powdered green tea gaining global popularity, is derived from the same plant as regular green tea (*Camellia sinensis*) but contains higher concentrations of catechins, particularly epigallocatechin gallate (EGCG, the main compound responsible for green tea’s antiplatelet effects). While it is theoretically expected to exert similar platelet-inhibitory properties, clinical evidence is currently lacking.

## Immune-mediated

Antibody-mediated platelet destruction is seen in the following diseases/conditions:

• Immune thrombocytopenia

Immune thrombocytopenia (ITP) is an acquired autoimmune disorder characterized by isolated thrombocytopenia with predisposition to mucocutaneous bleeding. The pathophysiology is still incompletely understood; however, the primary mechanism is the formation of autoantibodies directed against platelet surface antigens, which tag platelets for destruction by macrophages in the reticuloendothelial system of the spleen and/or liver. These antibodies also impair platelet production by megakaryocytes. Additional proposed mechanisms include impaired immune regulation, such as reduced regulatory T-cell activity and heightened cytotoxic CD8 + T-cell responses, which contribute to direct platelet lysis and impaired thrombopoiesis (26, 27). Bleeding phenotype generally correlates with the extent of thrombocytopenia; in fact, severe thrombocytopenia (i.e., platelet < 20 × 10^9^/L) is the strongest independent predictor of bleeding in ITP (28, 29). Other factors that further increase the bleeding risk include a previous history of bleeding and age over 60 years (29). Paradoxically, ITP is also associated with an increased thrombotic risk, particularly for venous thromboembolism (VTE), with rates estimated to be approximately twice those of the general population (30, 31). Contributing mechanisms include platelet activation by the pathological antibodies and the release of procoagulant platelet microparticles (PMPs), which can express tissue factor and propagate thrombin generation. The use of thrombopoietin receptor agonists further augments this risk. Finally, the extensive interplay between platelets, the coagulation system, and the complement system can exacerbate the concurrent bleeding and thrombotic tendencies observed in ITP (32).

• Acquired autoimmune platelet function disorders due to inhibitory antibodies

In rare instances, autoantibodies targeting specific platelet glycoproteins can exhibit functional inhibitory activity, resulting in qualitative platelet dysfunction. This leads to clinically significant mucocutaneous bleeding that does not correlate with the degree of thrombocytopenia, as generally seen in ITP patients. The autoantibodies can be directed against the αIIbβ3 integrin (GPIIb/IIIa) or the GPIb-IX-V complex, leading to acquired Glanzmann thrombasthenia or acquired Bernard-Soulier syndrome, respectively (33). They can also be directed against other antigens, such as GPIV (34). These conditions can occur in the background of hematological neoplasms (e.g., lymphoproliferative disorders) or complicate other autoimmune disorders, including ITP. Such antibody-mediated platelet dysfunction should be strongly considered in patients presenting with new onset disproportionate bleeding severity relative to the platelet count, particularly when conventional coagulation studies remain normal (e.g., PT and aPTT). Characteristically, expected specialized testing usually demonstrates intact glycoprotein expression (e.g., by flow cytometry) with a defective function (e.g., by light transmission aggregation studies) (35, 36).

• Systemic lupus erythematosus

The common platelet defect seen in this category is hypercoagulability, primarily caused by abnormal immune-complex-mediated platelet activation via the Fcγ receptor IIA. This state of hypercoagulability is further increased in the presence of lupus anticoagulant (LA) and anticardiolipin antibodies (aCL), which can be seen in 5–34% and 12–44% of Systemic Lupus Erythematosus (SLE) patients, respectively (37, 38). Furthermore, around 10% of SLE patients are diagnosed with antiphospholipid syndrome with a more amplified thrombotic risk, especially in individuals who are triple positive for (LA, aCL and anti-β2 glycoprotein I) (38, 39). Thrombocytopenia is another well-recognized feature of SLE and constitutes one of the hematologic classification domains in the ACR/EULAR 2019 criteria (40). Similar to ITP, it is mainly immune-mediated, caused by autoantibodies against platelet surface antigens; however, these antibodies are immunologically distinct from those in ITP. The severity of thrombocytopenia often correlates with disease activity and tends to fluctuate over time. Less commonly, platelet dysfunction due to storage pool deficiency may occur, exacerbating the impact of thrombocytopenia (41). Interestingly, beyond these changes, an expanding body of evidence highlights that platelet-derived mediators and surface molecules can influence the pathogenesis of SLE and other autoimmune diseases like rheumatoid arthritis by modulating innate and adaptive immune responses (42). These immunomodulatory roles of platelets are further discussed in the inflammation section.

## Systemic diseases-associated platelet disorders

A wide range of systemic diseases can adversely affect platelet count and/or function and lead to either bleeding or thrombosis of varying severity, complicating the symptoms of the underlying disease, and might even be the most severe of them all. The mechanism by which platelets are affected is variable and depends on the underlying systemic disease. Some of the most common hematological and non-hematological disorders are discussed here.

• Hematological disorders

There is a wide range of hematological disorders that affect the platelets, from malignant disorders like acute leukemias to non-malignant diseases like thrombotic microangiopathies.

o Thrombotic microangiopathies

Thrombotic microangiopathies (TMAs) are a group of life-threatening disorders that encompass thrombotic thrombocytopenic purpura (TTP), hemolytic uremic syndrome (HUS), atypical hemolytic uremic syndrome (aHUS), and Hemolysis, Elevated Liver enzymes, Low Platelet count syndrome (HELLP syndrome). These syndromes are characterized by the triad of microangiopathic hemolytic anemia (MAHA), thrombocytopenia (TP), and organ dysfunction due to widespread microvascular platelet-rich thrombi (43). Profound TP is a hallmark of TMAs, and it primarily results from excessive platelet activation and consumption within the microvasculature. In TTP, this process is driven by a severe deficiency of ADAMTS13, a metalloprotease responsible for cleaving ultra-large von Willebrand factor (VWF) multimers that are highly thrombogenic. In the absence of ADAMTS13 activity, these multimers persist, promoting uncontrolled platelet adhesion and aggregation, which leads to the formation of widespread VWF- and platelet-rich microthrombi (44). Most TTP cases are immune-mediated, caused by acquired autoantibodies that inhibit or clear ADAMTS13. Congenital TTP (also known as Upshaw–Schulman syndrome), which is driven by pathogenic variants in ADAMTS13 gene, is rare, accounting for <5% of cases (45). In HUS, this occurs due to a microbial pathogen, most commonly a Shiga toxin-producing *Escherichia coli* (STEC) infection; other, less common infectious causes include *Streptococcus pneumoniae* and influenza virus (46). By contrast, aHUS results from dysregulated activation of the alternative complement pathway; the complement defect may be inherited (due to pathogenic variants in regulators or components, such as CFH, CFI, MCP/CD46, C3, and CFB) or acquired through autoantibodies, most commonly anti–factor H (47). HELLP syndrome probably represents a severe form of preeclampsia and can occur during pregnancy or the postpartum period (48). Although its pathophysiology is not yet fully understood, it likely arises from abnormal development of the placental vasculature with placental hypoxia, hypoperfusion, and ischemia, which drives excess maternal release of antiangiogenic factors and causes systemic endothelial dysfunction and activation, ultimately leading to platelet-rich microvascular thrombosis and other manifestations (49). A subset of HELLP cases appears to involve complement-mediated thrombotic microangiopathy (CM-TMA); this might have therapeutic implications- see management section (50).

Beyond quantitative loss, there is increasing evidence for qualitative platelet defects in TMAs. Platelets in TTP and related syndromes also exhibit abnormal activation states, reflected by increased surface P-selectin and excess circulating platelet–leukocyte aggregates, and they shed abundant platelet-derived microparticles; these microparticles are strongly procoagulant and are elevated during acute disease with levels that fall as patients improve, implicating them both as mediators of microvascular thrombosis and as potential markers of disease activity (44, 51). On the contrary, chronic exposure to ultra-large VWF multimers and ongoing endothelial injury can lead to platelet exhaustion and impaired function, further compounding the bleeding risk despite the prothrombotic state (52).

o Myeloproliferative neoplasms

Myeloproliferative Neoplasms (MPNs) are a heterogeneous group of clonal hematopoietic stem cell disorders characterized by an abnormal proliferation of one or more myeloid cell lines. MPNs can present with the full spectrum of platelet abnormalities, encompassing: thrombocytosis, a common finding in MPNs, particularly, chronic myeloid leukemia and essential thrombocythemia. Significant thrombocytosis (i.e., >1,000 × 10^9^/L) can paradoxically present with bleeding due to acquired von Willebrand syndrome (AVWS) caused mainly by adsorption of high-molecular-weight von Willebrand Factor antigens onto platelets (53); thrombocytopenia, which is usually seen in advanced stages (e.g., post polycythemia myelofibrosis, progression to acute leukemia), and is usually due to bone marrow occupation by fibrosis and/or blasts; platelet dysfunction with reported significant defects in platelet aggregation, granule content, surface glycoproteins, and fibrinogen binding; and platelet hyperactivity was also reported in patients with JAK2 mutation, which could be an important player in the pathogenesis of thrombosis, a common complication in MPNs (54). It is important to note that the platelet dysfunction detected by laboratory assays does not correlate with the bleeding phenotype in these patients, highlighting the multifactorial nature of bleeding in this context (55, 56).

o Myelodysplastic neoplasms

These are another heterogeneous group of clonal hematopoietic disorders. Bleeding can complicate the course of these patients and is usually due to a low platelet count with functional defects. Both alpha granule reductions and abnormalities in the dense tubular system were reported (57). Thus, these patients usually show a bleeding phenotype more severe than the extent of thrombocytopenia (58).

o Acute leukemia and other malignant bone marrow infiltration disorders

Thrombocytopenia is common in acute leukemia and other hematologic malignancies and is a major driver of bleeding and early mortality; in a large cohort, ~28% of adults with hematologic cancers had thrombocytopenia (59). Importantly, low platelet counts do not fully explain the bleeding risk in these disorders; qualitative platelet dysfunction and consumptive coagulopathies, including disseminated intravascular coagulation (DIC) also contribute substantially (60).

Acute leukemia can impact the bone marrow (BM) microenvironment and suppress normal hematopoiesis, including megakaryopoiesis (61). Evidence shows that leukemia suppresses the growth and maturation of megakaryocytes, contributing to thrombocytopenia during active disease (62).

Infiltration of the BM by abnormal blasts or non-hematological cells (e.g., metastasis) reduces megakaryocyte reserves and further depresses platelet production (55).

Additionally, peripheral platelet destruction, caused by consumptive coagulopathy (e.g., DIC) and/or cancer-associated coagulation, further aggravates thrombocytopenia (63).

Independent of thrombocytopenia, acute leukemia is associated with platelet function defects, as demonstrated by impaired platelet activation, adhesion, and aggregation. Flow cytometric and functional studies show reduced P-selectin (CD62P) and CD63, decreased GPIb (CD42b), and alterations in GPIIb/IIIa (CD61/CD41), respectively. Changes that correlate with bleeding and suggest a global signaling defect. Notably, platelet function tests (aggregation/activation) predict bleeding better than platelet count alone in acute leukemia with thrombocytopenia, reinforcing the clinical salience of qualitative defects (60).

o Other hematological disorders: paraproteinemia and lymphoproliferative disorders

Patients with paraproteinemia and lymphoproliferative disorders (LPD) can experience both quantitative and qualitative platelet defects. These complications are most common in malignant conditions like multiple myeloma, Waldenström macroglobulinemia, and chronic lymphocytic leukemia/lymphoma (CLL), but can also occur in pre-malignant conditions, such as monoclonal gammopathy of undetermined significance (MGUS). Their presence can lead to significant hemostatic complications, primarily bleeding (64).

Thrombocytopenia (TP) is multifactorial and can be caused by decreased platelet production due to displacement of megakaryocytes and suppression of thrombopoiesis (e.g., by abnormal space occupation of the bone marrow by malignant plasma cells) (65). Immune platelet destruction can also contribute to TP. In some LPD, mainly CLL, the clonal B-lymphocytes can produce autoantibodies (i.e., immune thrombocytopenia, ITP) that increase platelet clearance (66). Interestingly, ITP can either complicate the course of CLL or, in rare instances, precede its diagnosis (67). This may be suspected when there is a discrepancy between the platelet count and disease burden in the bone marrow. Splenic sequestration is frequently seen in LPD and is another well-established cause for TP (68).

Defective platelet function is caused mainly by the nonspecific binding of paraproteins to the platelet surface receptors, which blocks the platelet receptors and prevents normal adhesion and aggregation. Studies have shown that adding isolated paraproteins to healthy platelets can significantly impair their ability to aggregate in response to multiple agonists (69, 70). In Waldenström macroglobulinemia, high levels of IgM paraprotein increase blood viscosity, leading to sluggish blood flow. This physical change can interfere with and impair platelet function (71). Furthermore, paraproteins can bind to and accelerate the clearance of von Willebrand factor (VWF), leading to increased clearance and reduced levels (especially of the large molecular size multimers). This eventually can lead to acquired von Willebrand syndrome (55).

Beyond the mentioned platelet defects, paraprotein disorders can also significantly impair other components of the coagulation system, including acquired factor X deficiency and defective fibrin polymerization, which can further amplify bleeding risk (72–74). This extends beyond the scope of this review.

• Non-hematological disorders

o Liver disease

Both quantitative and qualitative platelet defects may be seen and can contribute to either a hypocoagulable or hypercoagulable state in individuals with liver disease. Thrombocytopenia (TP) is commonly seen in chronic liver disease, and its prevalence varies from 6% in chronic hepatitis to up to 78% in patients with cirrhosis (8). It primarily results from decreased hepatic thrombopoietin production, with its severity correlating with the extent of liver disease. Hypersplenism can also contribute to the extent of TP; with the advancement of liver disease, there is a gradual reduction in the diameter of the hepatic vascular bed, accompanied by an increase in splanchnic inflow, leading to increased pressure in the portal vein, splenomegaly, and subsequent platelet sequestration and TP. Immune-mediated platelet clearance/destruction is another major contributor to TP. Antiplatelet antibodies (such as anti-GPIb-IX-V and anti-αIIbβ3) are significantly elevated in chronic liver disease, with their titres showing an inverse correlation to platelet counts (75). Additional contributing factors include bone marrow suppression from viral infections (e.g., hepatitis B or C), adverse effects of antiviral therapy, and infiltration by hepatocellular carcinoma (76). In the majority of cases, TP is often mild, and spontaneous bleeding is uncommon unless platelet counts fall below 10–20 × 10^9^/L (24).

Beyond TP, patients with liver disease often exhibit qualitative platelet dysfunction. Two cyclic nucleotides, cyclic adenosine Monophosphate (cAMP) and cyclic guanosine monophosphate (cGMP), are key intracellular inhibitors of platelets. They exhibit their inhibitory action by reducing intracellular calcium levels and downregulating integrin αIIbβ3 activity, thus impairing aggregation. In portal hypertension, endothelial dysfunction results in a reduction in the synthesis of prostacyclin (PGI₂) and nitric oxide (NO), both of which are key inhibitors of platelet activation (77). They exert their effects by increasing intracellular levels of cyclic nucleotides. This leads to platelet hyperactivity. Additional contributors to the hypercoagulable platelet state include elevated levels of von Willebrand factor (VWF), which increases platelet adhesion abilities, increased platelet-derived microparticles, and enhanced platelet responsiveness to various agonists (78).

On the contrary, platelet hypocoagulability can also be observed in patients with liver disease (79), which is driven by several extrinsic factors that negatively impact transmembrane signaling, secretion, and aggregation. In the hyperdynamic circulation, frequently encountered in cirrhosis, endothelial overproduction of NO/PGI₂ suppresses platelet activation and contributes to *ex vivo* hyporeactivity. This pathway is opposite to what was previously discussed in cases with liver disease and endothelial dysfunction (77, 80). Portal inflammation causes *in vivo* platelet activation (higher soluble P-selectin and soluble GPVI in the portal vs. systemic blood), supporting a cycle of pre-activation/ “platelet exhaustion.” Supporting the “platelet exhaustion” concept, platelets from cirrhotic individuals show depleted dense (ATP and serotonin) and alpha (PF-4 and β-thromboglobulin) granule contents, with an increased plasma β-thromboglobulin: PF-4 ratio, indicating *in vivo* release of granule contents, with a net result of “exhausted” and hyporeactive platelets (81, 82). Finally, in cholestatic states, bile acids can directly inhibit platelet activation and adhesion, thereby reducing thrombus formation (83). These milieu effects align with the contemporary phenotype measured in cirrhosis: reduced aggregation to multiple agonists, reduced dense-granule ATP release, and suppressed activation (reduced P-selectin/CD62P expression and PAC-1 binding) as measured by flow cytometry (81).

These abnormalities contribute to bleeding risk, particularly in advanced liver disease. With that said, growing evidence suggests that most of these patients are, in fact, in a “hemostatic balance,” a concept elaborated upon by Lisman and Porte (84). Clinically, this is reflected by the ability of many patients with cirrhosis to undergo major surgical procedures without the need for prophylactic transfusion (85). However, this balance can be easily disrupted and shifted toward either bleeding or thrombotic events, especially with an added risk factor in either direction (3). Moreover, the most clinically significant bleeding episodes in cirrhosis, such as variceal hemorrhage, are primarily attributable to local vascular abnormalities and elevated splanchnic pressures, rather than intrinsic defects in the coagulation system (84). Furthermore, platelet function assessed via thrombin generation assays is often preserved (86). Although routine coagulation tests such as prolonged prothrombin time (PT) and activated partial thromboplastin time (aPTT) often suggest a hypocoagulable state, their clinical significance is limited. Notably, prolonged PT and INR do not accurately predict bleeding risk and should not be used in isolation to guide clinical decisions (85).

o Kidney disease/renal failure

Platelet dysfunction in kidney disease has a multifactorial basis, with uremia being a major cause of bleeding through direct impairment of platelet function. Uremic toxins interfere with platelet membrane receptors, reduce intracellular calcium mobilization, and impair granule secretion, leading to defective platelet adhesion, aggregation, and secretion even when platelet counts are normal (87). Other extrinsic factors that also impair platelet function include excess amounts of platelet inhibitors, such as prostacyclin (PGI_2_) and nitric oxide (NO), discussed previously, which increase intracellular cAMP. The net effect of these factors is demonstrated by defective platelet function, which includes diminished integrin response and platelet activation to stimulating agonists, reduced synthesis and release of thromboxane TXA_2_, deficiency of platelet dense granules, and decreased expression of surface receptors (88).

Anemia, a frequent finding in renal failure, also contributes to platelet dysfunction (89). Evident by reducing the rheological effect of red blood cells, anemia impairs platelet adhesion and aggregation at sites of vascular injury and enhances the inhibitory action of NO and PGI_2_, which would otherwise be partially neutralized by red cells. Treatment with erythropoietin can improve bleeding time, but it does not restore platelet activation, highlighting the multifactorial and complex nature of the platelet defect in this setting (90).

o Inflammation and sepsis

Inflammation is a complex immune response to harmful stimuli, such as pathogens, aimed at eliminating the threat, limiting tissue injury, and initiating repair. When this response becomes hyperactivated and dysregulated, it can progress to sepsis, a life-threatening condition characterized by multi-organ dysfunction. Platelets play pivotal roles not only in hemostasis but also in innate immunity, propagation of inflammation, and the pathogenesis of sepsis. Consequently, thrombocytopenia (TP) is a common finding in sepsis and serves as an established prognostic indicator of increased morbidity and mortality (91). TP in sepsis is primarily attributed to platelet consumption, notably via the formation of platelet–leukocyte aggregates. These complexes form when activated platelets express P-selectin, which binds to P-selectin glycoprotein ligand-1 (PSGL-1), a mucin-like glycoprotein expressed on the surface of leukocytes. This interaction promotes adhesion to the inflamed endothelium and amplifies local inflammation. It also induces the release of neutrophil extracellular traps (NETs) and upregulates monocyte tissue factor expression, linking immune activation to thrombus formation. More elaboration on the interplay between inflammation and thrombosis is discussed by Iba and Levy (91). Another factor leading to TP is excessive platelet activation triggered by elevated levels of von Willebrand factor (VWF), resulting from acquired ADAMTS-13 deficiency, a common finding in sepsis. This imbalance promotes intravascular platelet aggregation, contributing to microvascular thrombosis and organ dysfunction.

Unlike classical hematologic, immunologic, or drug-related causes of platelet dysfunction detailed in earlier sections, inflammation-driven platelet activation and dysfunction is often underrecognized despite being despite being common in practice and clinically significant. For instance, TP occurs in approximately 50% of patients with severe sepsis or septic shock (92). Finally, the immunomodulatory role of platelets has emerged as a significant area of interest over the past two decades, highlighting their involvement in both the initiation and amplification of systemic inflammation and further solidifying their relevance in the pathophysiology of sepsis (93).

## Disorders of abnormal circulatory shear and hemodynamic stress

Conditions like extracorporeal membrane oxygenation (ECMO), Cardiopulmonary Bypass (CPB), and cardiac prosthetic devices cause significant quantitative and qualitative platelet defects due to a plethora of causes, including a significantly disturbed circulation related to abnormal flow, extensive exposure to artificial surfaces, massive cellular activation, hypothermia, shear stress, and the use of exogenous drugs (heparin and protamine). Beyond the extensive effect on platelets, a complex series of steps leads to a thromboinflammatory state involving thrombosis, inflammation, and innate immunity that extends beyond the scope of this review. Thrombocytopenia (TP) can complicate >30% of cardiac surgeries and is an independent factor in postoperative morbidity and mortality (94). TP results mainly from hemodilution, direct mechanical destruction, and the huge inflammatory state. The requirement for systemic anticoagulation places these patients at additional risk for developing heparin-induced thrombocytopenia (HIT) that can complicate 3.7% of ECMO in adults (95, 96). HIT is a serious, immune-mediated adverse reaction to heparin polyanions caused by pathogenic immunoglobulin G (IgG) antibodies. IgGs bind to complexes of heparin and PF4 (97). These immune complexes bind platelets via their surface receptors FCγIIa, leading to massive activation and a switch toward a hypercoagulable state. High-speed centrifugation can trigger receptor shedding, impairing adhesion and aggregation, and can also cause fragmentation; these changes can compromise platelet count and function and lead to bleeding complications. On the contrary, significant platelet activation, starting mainly by contact with artificial surfaces, leads to aggregation, release of procoagulant microparticles, and thrombotic complications (98).

A summary of the causes of platelet dysfunction is shown in Table 3.

**Table 3:** Summary of causes of acquired platelet disorders.

Category	Specific cause	Net effect	Mechanism
Drug-induced^*^	Aspirin	Qualitative ↓	Irreversible COX-1 inhibition → ↓ TXA₂ → impaired aggregation; effect lasts lifespan of platelets (7–10 d)
	αIIbβ3 antagonists (abciximab, tirofiban, eptifibatide, zalunfiban^¶^)	Qualitative ↓ Quantitative ↓ (rare)	Block integrin αIIbβ3 → inhibit aggregation to all agonists Rare immune-mediated TP due to drug-dependent antibodies
	NSAIDs (ibuprofen, naproxen, indomethacin)	Qualitative ↓	Reversible COX-1 inhibition → ↓ TXA₂ → “transient” impaired aggregation; (~24 h)
Herbals/supplements^*§^	*Ginkgo biloba*, others	Qualitative ↓	Inhibit AA–induced aggregation; synergistic with aspirin. Evidence inconsistent, mostly preclinical
Alcohol	Acute ingestion	Qualitative ↓	Impaired aggregation to several agonists Impaired arachidonic acid mobilization, ↓ TXA₂ → impaired aggregation ↓ αIIbβ3 expression
	Later phase	Qualitative ↑	↓ cAMP restraint “transient” → downstream activation ↑ P-selectin & platelet–neutrophil complexes ↑ platelet aggregation with thrombin
	–	Quantitative ↓^‖^	Myelosuppression, direct platelet toxicity, ↑ apoptosis, ↓ TPO
	–	↑MPV	Reticulated platelets
Immune-mediated	ITP	Quantitative ↓ Qualitative ↑	↑ Clearance, impaired thrombopoiesis, impaired immune regulation ↑ Activation and release of procoagulant microparticles → paradoxical thrombosis risk
	“Functional” Inhibitory autoantibodies (acquired GT, acquired BSS)	Qualitative ↓	Autoantibodies “functionally” block GPIIb/IIIa or GPIb/IX/V complex → defective aggregation or adhesion
	SLE	Quantitative ↓ Qualitative ↑ Qualitative ↓	Autoantibody-mediated TP Immune complex-mediated platelet activation, LA, aCL, aβ2GPI → thrombosis Storage pool deficiency
Systemic diseases (hematological)	TMA (TTP, HUS, aHUS, HELLP)	Quantitative ↓ Qualitative ↑ Qualitative ↓	(Hallmark): consumptive; PLT-rich microvascular thrombi TTP: ADAMTS13 deficiency^†^ →↑ UL-VWFM→ widespread PLT activation HUS: Shiga toxin/endothelial damage aHUS: complement dysregulation → endothelial injury HELLP: Abnormal development of placental vasculature (early in pregnancy) → placental hypoxia/hypoperfusion/ischemia→ anti-angiogenic factors → endothelial dysfunction/activation ↑ P-selectin, ↑ circulating platelet–leukocyte aggregates, ↑ procoagulant microparticles (chronic) exposure to UL-VWFM & endothelial injury→ platelet exhaustion
	MPN	Quantitative ↑/↓ Qualitative ↑/↓	Thrombocytosis: clonal hematopoiesis → uncontrolled thombopoiesis TP: fibrosis/leukemic transformation Defected aggregation, ↓ surface GP expression, ↓ granule content and fibrinogen binding JAK2 mutation → hyperactive platelets
	MDS	Quantitative↓ Qualitative ↓	Clonal hematopoiesis→ thrombocytopenia Clonal hematopoiesis granule defects (α-granules, dense tubular system)
	Acute leukemia/BM infiltration	Quantitative ↓ Qualitative ↓	Suppressed megakaryopoiesis, BM space occupation, consumptive coagulopathy ↓ CD62P/CD63, ↓/defected surface GP expression
	Paraproteinemia/LPD	Quantitative ↓ Qualitative ↓	Marrow displacement, ITP, splenic sequestration Direct paraprotein binding to GP → blocking normal function ↑ viscosity (IgM) → defected flow→ impaired PLT function
Systemic diseases (non-hematological)	Liver disease	Quantitative ↓ Qualitative ↑/↓	↓ TPO, splenic sequestration, ITP, marrow suppression ↑ cAMP/cGMP → ↓ aggregation Hyperdynamic circulation→ endothelial dysfunction → PLT suppression
	Renal failure (uremia)	Qualitative ↓	Uremic toxins → impaired receptors, ↓ Ca^2+^ mobilization, ↓ secretion. ↑PGI₂/NO → ↑cAMP→ ↓ aggregation Anemia (loss of RBC rheological effect) → ↓ adhesion/aggregation
	Inflammation/sepsis	Quantitative ↓ Qualitative	Consumption in platelet–leukocyte aggregates, ↑ VWF + ADAMTS13 deficiency → ↑ PLT activation/aggregation →microthrombosis
Mechanical/shear stress	ECMO, CPB, prosthetic devices	Qualitative ↑/↓ Quantitative ↓	Heparin use→ HIT→PLT activation/clearance, High-speed centrifuges: receptor shedding → impaired PLT function Artificial surface contact →PLT activation/aggregation/release of procoagulant microparticles Hemodilution, direct mechanical destruction, hyperinflammatory state, Hypothermia, shear stress

AA, arachidonic acid; aβ2GPI, anti-β2 glycoprotein I; aCL, anticardiolipin antibodies; aHUS, atypical hemolytic uremic syndrome; BM, bone marrow; BSS, Bernard–Soulier syndrome; COX-1, cyclooxygenase-1; CPB, cardiopulmonary bypass; DIC, disseminated intravascular coagulation; ECMO, extracorporeal membrane oxygenation; GP, glycoproteins; GT, Glanzmann thrombasthenia; HELLP, Hemolysis, Elevated Liver enzymes, Low Platelet count; HIT, heparin-induced thrombocytopenia; HUS, hemolytic uremic syndrome; ITP, immune thrombocytopenia; LA, lupus anticoagulant; LPD, lymphoproliferative disorders; MDS, myelodysplastic syndromes; MPN, myeloproliferative neoplasms; MPV, mean platelet volume; NETs, neutrophil extracellular traps; NSAIDs, nonsteroidal anti-inflammatory drugs; PLT, platelets; SLE, systemic lupus erythematosus; TMA, thrombotic microangiopathies; TP, thrombocytopenia; TPO, thrombopoietin; TTP, thrombotic thrombocytopenic purpura; TXA_2_, thromboxane A2; UL-VWFM, ultra-large von Willebrand factor multimers; VWF, von Willebrand factor.

^¶^Not FDA-approved yet; ongoing Phase 3: (CELEBRATE trial. ClinicalTrials.gov NCT04825743).

^*^Other drugs with their inhibitory effects are summarized in Tables 1, 2, respectively.

^§^Most of the available evidence is preclinical and shows inconsistencies across studies.

^‖^Genetic predisposition might impact the extent of thrombocytopenia.

^†^Can be acquired due to autoantibodies or congenital.

## Evaluation

Evaluating patients with suspected acquired platelet disorders can be challenging, as bleeding manifestations are often subjective, and more importantly, in cases with underlying chronic illnesses and multiple comorbidities, dedicated workup for acquired platelet dysfunction is not often carried out, contributing the bleeding directly to the disease. Additionally, many of the available laboratory evaluations are limited when the platelet count is low, which is common in these individuals, as thrombocytopenia itself may confound test results; recent studies have sought to standardize such testing in low-count samples (99). Moreover, some assessments still rely on classic coagulation parameters, such as prothrombin time (PT) and activated partial thromboplastin time (aPTT), which have poor predictive value for bleeding risk in several settings, particularly in liver disease (7, 85).

**Figure 1:**
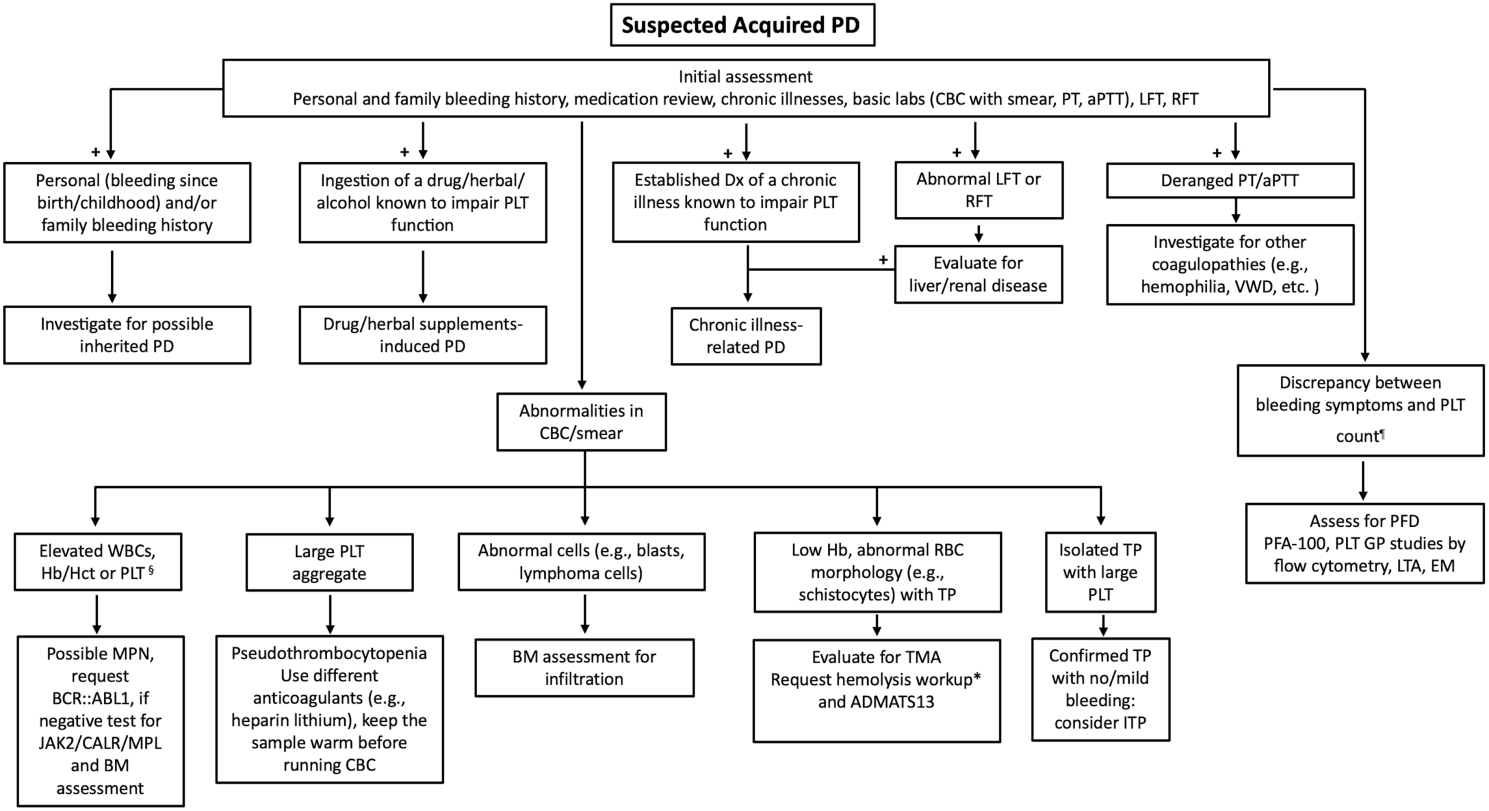
Diagnostic algorithm for suspected acquired platelet dysfunction. ADAMTS13, a disintegrin and metalloproteinase with thrombospondin type 1 motif, member 13; aPTT, activated partial thromboplastin time; BCR::ABL1, BCR–ABL1 fusion (Philadelphia chromosome); BM, bone marrow; CALR, calreticulin; CBC, complete blood count; Dx, diagnosis; EM, electron microscopy; GP, Glycoprotein; Hb, hemoglobin; Hct, hematocrit; ITP, immune thrombocytopenia; JAK2, Janus kinase 2; LFT, liver function test; LFT, liver function test; LTA, light transmission aggregometry; MPL, thrombopoietin receptor gene; MPN, myeloproliferative neoplasms; PD, platelet defects; PFA-100, Platelet Function Analyzer-100; PFD, platelet function defect; PLT, platelet; PT, prothrombin time; RBC, red blood cells; RFT, renal function test; TMA, thrombotic microangiopathies; TP, thrombocytopenia; VWD, von Willebrand disease; WBC, white blood cells. § Ruling out reactive causes is needed. *Hemolysis workup: corrected reticulocytes, indirect bilirubin, direct Coombs test, LDH and haptoglobin. ¶ Either bleeding despite normal platelet counts, or disproportionate bleeding to the degree of platelet reduction.

While standardized bleeding assessment tools (BAT) such as the ISTH-BAT have proven valuable in clinical practice, especially in von Willebrand disease, a significant limitation exists as these tools are relatively insensitive and have not been validated for acquired platelet defects. Consequently, the clinical assessment must emphasize alternative diagnostic indicators specific to acquired platelet dysfunction, including detailed medication histories (particularly antiplatelet agents, anticoagulants, and herbal supplements), recent hematological or systemic diagnoses, etc. (100). Figure 1 illustrates a modified algorithmic approach that incorporates these considerations for individuals with suspected acquired platelet defects.

### Laboratory evaluation

• Complete blood count and platelet morphology

The initial laboratory evaluation of platelets (PLT) begins with quantifying the PLT count. It is essential to confirm any reported thrombocytopenia, especially when observed for the first time. One important consideration is pseudothrombocytopenia, an *in vitro* phenomenon that occurs in approximately 0.03–0.3% of cases and is most commonly induced by the use of ethylenediaminetetraacetic acid (EDTA) as an anticoagulant (101). If unrecognized, this phenomenon can lead to extensive and unnecessary diagnostic workup or inappropriate therapeutic interventions. In high-throughput laboratories processing around 2,000 samples daily, this could translate to 1–6 cases of pseudothrombocytopenia per day. The Mean Platelet Volume (MPV) is another readily available parameter from the complete blood count (CBC). It is very helpful in approaching persons with acquired PLT defects when interpreted in the context of clinical presentation. For instance, elevated MPV often reflects increased platelet turnover, as seen in immune thrombocytopenia (102). However, other entities, such as myelodysplastic syndrome, can also lead to an abnormally large PLT. Another key parameter, relatively newly introduced to automated analyzers, is the fraction of immature platelets (IPF). IPF is a parameter that measures the fraction of young platelets released into the circulation based on their RNA content using specific fluorescence techniques. Thus, it is an informative tool that reflects thrombopoiesis; it is increased in cases with peripheral destruction (i.e., immune thrombocytopenia), and classically decreased in cases with impaired thrombopoiesis (i.e., bone marrow infiltration by acute leukemia) (64).

• Platelet function assessment:

1. Platelet function assays (PFA-100) is a relatively automated automated test performed on citrated whole blood. It measures the closure time (CT), which reflects the duration required for a platelet plug to occlude a microscopic aperture within a membrane coated with platelet agonists (collagen with either epinephrine or ADP) under high shear conditions that simulate the physiological conditions of blood vessels. PFA-100 is sensitive to moderate–severe platelet function defects, including significant acquired platelet dysfunctions. However, its specificity is limited, as prolonged CT may also result from conditions such as von Willebrand disease. Importantly, since the assay is performed on whole blood, reduced platelet counts and/or hematocrit may yield prolonged CTs unrelated to true platelet dysfunction. Thus, the PFA-100 is not a reliable tool in patients with thrombocytopenia, and normal results do not exclude mild platelet function defects (103).
2. Flow cytometry: The most commonly used test is the quantification of platelet surface antigens using monoclonal antibodies on freshly collected samples (e.g., CD41 against integrin αIIb and CD61 against integrin β3), which can be markedly low in cases of acquired Glanzmann thrombasthenia (33). For this test, fresh samples should be available. Advantages include the ability to evaluate platelets even in samples with low platelet counts. Flow can also be used to measure platelet activation using monoclonal antibodies against surface antigens, such as P-selectin (CD62P), or activated surface receptors, like the activated form of αIIbβ3 (PAC-1). Another area that can be assessed by flow is the evaluation of agonist-induced secretion, such as the release of dense granule contents, using CD63, mepacrine, and LAMP-2 to detect acquired dense granule deficiency, which can be observed in various diseases, including myeloproliferative and myelodysplastic neoplasms. Furthermore, flow cytometry serves multiple other roles in assessing platelet function, including the evaluation of platelet-leukocyte aggregates, the detection of platelet-derived microparticles, and the monitoring of the efficacy of antiplatelet therapy. This has been extensively elaborated upon by Jourdi et al. (104).
3. Light transmission aggregometry (LTA) is considered the gold standard for evaluating platelet function. It studies the dynamics of platelet aggregation *in vitro* in the presence of a panel of agonists in platelet-rich plasma. However, despite its utility, it shows poor sensitivity for dense granule deficiency, a commonly acquired platelet defect. LTA can also be used to identify clopidogrel or aspirin resistance, thus contributing significantly to guiding appropriate antiplatelet regimen before interventional therapy (105).
4. Transmission electron microscopy (TEM) is the gold standard for evaluating platelet dense granule content, allowing direct visualization and enumeration of dense granules as electron-dense bodies within the platelet cytoplasm without the need for special staining. It is particularly valuable in diagnosing storage pool deficiencies, including acquired dense granule deficiency in systemic disorders. Confirmed abnormalities on TEM are highly predictive of an underlying bleeding disorder. However, it is not widely available and requires specialized expertise (106).
  While other platelet function tests, such as lumiaggregometry, impedance aggregometry, and global hemostasis assays like thromboelastography (TEG) and rotational thromboelastometry (ROTEM), provide valuable insights, especially in perioperative or intensive care settings, they are not routinely used for the diagnosis of acquired platelet dysfunctions. The tests presented here are selected for their clinical applicability, diagnostic value, and practicality in evaluating persons with acquired platelet defects. A more detailed discussion on advanced methodologies can be found in reviews by Michelson (107).

## Management of acquired platelet disorders

The management of acquired platelet dysfunction begins with identifying and addressing the underlying cause, whether it is related to medications, herbal supplements, or a systemic illness. In parallel, adjunctive therapies play a critical role in the management of non-severe mucocutaneous bleeding: desmopressin (DDAVP) reduces bleeding in uremia, aspirin-induced platelet dysfunction, and in cases of acquired mild storage pool deficiencies by enhancing the release of von Willebrand factor (VWF) and promoting platelet adhesion (108); tranexamic acid is an antifibrinolytic agent that reduces mucosal bleeding in dental procedures or menorrhagia; and local hemostatic measures like fibrin sealants and topical thrombin. These interventions are particularly valuable for dental procedures and minor surgical interventions. Platelet transfusion is typically reserved for major bleeding or before emergency/urgent procedures. However, the effectiveness of transfused platelets may be limited in certain contexts (e.g., uremia, inhibitory effect of circulating antibodies), where adjunctive therapies such as desmopressin or dialysis may be more effective (109). The following sections will address disease/condition-specific management strategies, including targeted approaches. *Bleeding associated with antiplatelet therapy (APT)*: Although commonly encountered in clinical practice, managing bleeding in patients receiving APT remains a clinical challenge, and most available evidence is primarily based on expert opinion (110). Essential factors that guide management include the severity and location of bleeding, the time since the last dose was taken, and the patient’s additional risk of bleeding and/or thrombosis. Interruption of APT should only be undertaken after a careful evaluation of the competing risks: thrombosis if withheld and bleeding if maintained. The challenge extends beyond the recovery of bleeding. It extends to choosing the ideal timing for resuming the APT, as delays or premature re-initiation can expose patients to thrombotic and hemorrhagic risks, respectively (111). Initial management should include local measures to stop bleeding (surgery, compression, embolization, etc.); supportive measures like prevention of hypothermia and use of vasopressors; and transfusion support (e.g., plasma, specific factor replacement, and packed red blood cells). Early use of tranexamic acid is also effective in reducing bleeding (112, 113). If the initial approach measures were insufficient to control bleeding, platelet transfusion remains the principal modality to neutralize the effects of APT and can be used in bleeding with aspirin or thienopyridines (clopidogrel and prasugrel). However, circulating APT can substantially undermine the function and effectiveness of the transfused platelets (114). Of note, platelet transfusion should be avoided in cases of non-traumatic intracranial hemorrhage that does not necessitate surgical intervention in aspirin-treated patients, as it was associated with a higher mortality (115). Unlike Thienopyridines, ticagrelor is a direct and active inhibitor of P2Y_12_ that can neutralize transfused platelets for up to 24 h after ingestion. Accordingly, platelet transfusion cannot neutralize the effect of ticagrelor in the case of bleeding that occurs within 24 h of ingestion, with no available evidence on the efficacy of transfusing higher platelet doses. If ticagrelor was taken beyond 24 h, platelet transfusion could provide partial recovery (114). Bentracimab (MEDI2452) is an investigational recombinant monoclonal antibody that reverses the effect of ticagrelor and its active metabolite. It showed immediate and sustained reversal in randomized Phase 1/2b studies (116). This was further supported by an interim analysis of the pivotal Phase 3 REVERSE-IT trial, which showed rapid restoration of platelet function and >90% effective hemostasis in ticagrelor-treated patients requiring urgent surgery/procedure or presenting with major bleeding (117). Final Phase 3 results, presented at ACC 2025, confirmed rapid and durable reversal with high rates of adjudicated hemostasis (~83% in major bleeding), and regulatory approval is pending. While perioperative and periprocedural handling of antiplatelet agents is complex and requires a personalized approach, it has been comprehensively addressed in previous reviews by Godier et al. (118, 119). This topic extends beyond the scope of this review. *Immune Thrombocytopenia (ITP)*. As the course of ITP differs significantly between pediatrics and adults, with spontaneous remission being the usual course in pediatrics, this warrants an initial period of close monitoring. On the other hand, the disease course in adults tends to progress to chronic ITP, necessitating multiple lines of therapy. This includes corticosteroids, intravenous immunoglobulin (IVIG), anti-D immune globulin, rituximab, a monoclonal anti-CD20 antibody, thrombopoietin receptor agonists (TPO RA) (refer to Table 4), and splenectomy (26, 120). Fostamatinib is a relatively new orally administered medication that works by inhibiting spleen tyrosine kinase (Syk). Syk is a key signaling protein in the Fcγ receptor pathway involved in phagocytosis of antibody-coated platelets by splenic macrophages. Fostamatinib is FDA-approved for adult persons with chronic ITP who showed inadequate response to at least one prior therapy (121). Building on this approach, Rilzabrutinib, a new oral, reversible Bruton Tyrosine Kinase inhibitor, has recently been FDA-approved for adult ITP patients who did not show a sufficient response to previous therapies. In the Phase 3 LUNA-3 trial, rilzabrutinib achieved superior, durable, and more rapid platelet responses vs. placebo, with reduced rescue therapy/bleeding scores, while maintaining a favorable safety profile (122). Platelet transfusions are generally reserved for severe bleeding or before urgent or emergency procedures, although the responses are often attenuated due to the inhibitory effect of circulating antibodies on the transfused platelets. *Acquired Glanzmann thrombasthenia*: Management involves a multi-faceted approach targeting both acute bleeding control and long-term autoantibody suppression. For acute bleeding episodes, recombinant activated factor VII (rFVIIa) is often the preferred therapy. Platelet transfusions are reserved for non-responding patients or those with severe hemorrhage, although their efficacy can also be compromised by circulating autoantibodies. HLA-matched, single-donor, leukocyte-depleted apheresis platelets are preferred to minimize alloimmunization risk. Other adjunctive Hemostatic Measures mentioned earlier can be beneficial in less severe cases. Regarding the suppression of autoantibodies, corticosteroids remain the cornerstone and can be combined with other agents, such as azathioprine, rituximab, and IVIG, though responses can be variable and sometimes transient (123). *Thrombotic Microangiopathies (TMAs)*: Immediate recognition and management are critical due to the high mortality risk (43). The mainstay of therapy for thrombotic thrombocytopenic purpura (TTP) is urgent therapeutic plasma exchange (TPE), which removes autoantibodies and replenishes the ADAMTS13 enzyme. Adjunctive corticosteroids are recommended to suppress the production of autoantibodies. Recent advances include the addition of rituximab, which further reduces relapse rates. Caplacizumab, a nanobody targeting the A1 domain of VWF, blocks the interaction between VWF and platelets, rapidly controlling microvascular thrombosis and accelerating platelet count recovery. Caplacizumab is now recommended as part of initial therapy in acute episodes, in conjunction with TPE, and it shows the potential of eliminating the need for TPE in selected TTP patients (124, 125). On the other hand, the much less common congenital TTP (cTTP) has long been handled with simple plasma infusions to replace the missing ADAMTS13 (126). A major step forward is the recent development of an intravenous recombinant ADAMTS13 (apadamtase alfa; ADZYNMA), which has shown strong control of events and restoration of ADAMTS13 activity and is now approved for prophylactic and on-demand treatment in children and adults with cTTP (127). In the latest ISTH guidance, the panel supports ADAMTS13 replacement, when available, given its benefit–risk profile, and the use of fresh-frozen plasma when it is not (126). Decisions should be shared with patients, weighing benefits, harms, and practical burden. While the mainstay of therapy for typical hemolytic uremic syndrome (HUS) is best supportive care. As for the atypical HUS, plasma exchange was the historical standard, but the advent of eculizumab, a monoclonal antibody that inhibits terminal complement activation (C5), and the more recent, ravulizumab, a next-generation long acting C5 inhibitor, has revolutionized management and dramatically improved outcomes (128). The definitive therapy for hemolysis, elevated liver enzymes, low platelet count (HELLP) syndrome is immediate delivery of the fetus and placenta, which halts disease progression and is associated with improved maternal outcomes. Supportive measures for both the mother and fetus are paramount (129). Complement dysregulation may drive the thrombotic microangiopathy in a subset of HELLP patients. In a single case, eculizumab led to clinical and biochemical improvement and allowed a 17-day prolongation of pregnancy (50). Nevertheless, current practice guidelines for HELLP emphasize stabilization and delivery, and C5 inhibition is not recommended yet for management and should be reserved for confirmed complement-mediated TMA/aHUS after excluding TTP and other causes (130). *Paraproteinemia*: The main platelet defect is caused by the nonspecific binding of the paraprotein to the platelet surface receptors, and platelet transfusion has a limited clinical benefit. Platelet function improvement is generally achieved by the removal of the paraprotein from the circulation. This is also the main effective therapy for individuals who develop acquired von Willebrand Syndrome (AVWS). In settings when the paraprotein cannot be eliminated, intravenous gamma globulin infusions can prolong VWF survival and increase VWF levels. The addition of DDAVP or concentrates of von Willebrand factor (VWF) may be necessary depending on the severity of bleeding. Given the discussed mechanism, the clinical benefit will last for a short period before rebounding due to the same underlying persistent mechanism (3). *Alcohol-related thrombocytopenia (TP)*: management depends on the severity of TP and bleeding manifestations. More attention should be paid to preventing complications rather than treating TP, including avoiding the use of non-steroidal anti-inflammatory drugs (NSAIDs), optimizing dental care, hormonal therapy for menstrual disorders, and careful preoperative planning. It is also appropriate to monitor platelet counts after cessation (22). *Liver disease-related TP*: In patients with severe TP undergoing invasive procedures, platelet transfusion is the mainstay of therapy. However, several factors, including the short duration of the increment, the risk of adverse transfusion reactions, and the development of alloimmunization limit its use. Although the general threshold for transfusion is usually < 20 × 10^9^/L, growing evidence supports a lower threshold of 10 × 10^9^/L. A higher cut-off (i.e., 50–100 × 10^9^/L) should be chosen in settings with high bleeding risk (patient-related, disease-related, or procedure-related risks) (8). *Renal failure-related platelet defect*: Correction of anemia conventionally by erythropoietin and less commonly by transfusion was shown to decrease uremic bleeding (131). Additionally, dialysis corrects uremic platelet dysfunction by removing guanidinosuccinic acid and phenolic toxins from the circulation, improving platelet function and clot stability. Desmopressin can improve platelet function and bleeding symptoms (108). *Heparin-induced thrombocytopenia (HIT)*: Therapeutic anticoagulation with an alternative parenteral anticoagulant to heparin, such as argatroban, bivalirudin, danaparoid, and fondaparinux, should be administered in the acute phase of HIT, and depending on the presence of thrombosis, treatment should be continued for at least 4 weeks with a platelet count of greater than150 × 10 9 /L, if no thrombosis occurs, and for 3 months for cases of HIT with thrombosis. Notably, any form of heparin should be avoided, including heparin flushes, when HIT is suspected or confirmed. Patients can be switched to warfarin after the acute phase of HIT; after the platelet count has normalized or returned to baseline. At least 5 days of concurrent parenteral therapy are needed during transition. Care should be taken when switching from direct thrombin inhibitors (argatroban and bivalirudin) to warfarin due to INR elongation. Persons with stable HIT can be transitioned from parenteral anticoagulants to direct oral anticoagulants (DOACs), beyond the acute phase of the disease (97, 132).

**Table 4:** List of the uses of thrombopoietin receptor agonists.

TPO RA	Comments	Indicated use
Romiplostim	Sustained platelet responses >50 × 10^9^/L with reduced bleeding and improved quality of life in up to 80% of patients with chronic ITP (151) Although concerns have been raised about AML progression (152, 153), pooled data do not support a causal association (154, 155)	Chronic ITP (patients older than 1 year) refractory to other treatments (156, 157) Low/intermediate-1 risk MDS with TP^¶^
Eltrombopag	Theoretical risk of leukemic progression (152, 153) has not been confirmed in meta-analyses (154, 155)	Chronic ITP (patients older than 1 year) refractory to other treatments (158, 159) Low-risk MDS with severe or life-threatening TP^¶^ (160) HCV-associated TP: Previously used to facilitate interferon-based therapy; this indication has become largely obsolete with the shift to direct-acting antivirals (161)
Avatrombopag		Chronic ITP in adults (25) TP in adults with chronic liver disease scheduled to undergo a procedure (162)
Lusutrombopag		TP in adults with chronic liver disease scheduled to undergo a procedure (163)

AML, acute myeloid leukemia; HCV, hepatitis C virus; Ig, immunoglobulins; ITP, immune thrombocytopenia; SAA, severe aplastic anemia; MDS, myelodysplastic neoplasms; TP, thrombocytopenia; TPO, thrombopoietin; TPO RA, thrombopoietin receptor agonist.

^¶^This indication is off-label and not currently FDA-approved.

## Summary

Acquired platelet defects are commonly encountered across a broad spectrum of clinical settings, ranging from the use of herbal remedies and dietary supplements to complications associated with malignant conditions. Their impact varies from being an incidental finding of low platelet count to a severe, life-threatening bleeding/thrombosis. While addressing the underlying etiological factor is the cornerstone of treatment, several other therapeutic strategies may be warranted based on the bleeding severity, comorbid conditions, and procedural risk.
